# Working Memory Decline in Alzheimer’s Disease Is Detected by Complexity Analysis of Multimodal EEG-fNIRS

**DOI:** 10.3390/e22121380

**Published:** 2020-12-06

**Authors:** David Perpetuini, Antonio Maria Chiarelli, Chiara Filippini, Daniela Cardone, Pierpaolo Croce, Ludovica Rotunno, Nelson Anzoletti, Michele Zito, Filippo Zappasodi, Arcangelo Merla

**Affiliations:** 1 Institute for Advanced Biomedical Technologies, Department of Neuroscience and Imaging, University G. D’Annunzio of Chieti-Pescara, Via Luigi Polacchi 13, 66100 Chieti, Italy; antonio.chiarelli@unich.it (A.M.C.); chiara.filippini@unich.it (C.F.); d.cardone@unich.it (D.C.); pierpaolo.croce@unich.it (P.C.); f.zappasodi@unich.it (F.Z.); arcangelo.merla@unich.it (A.M.); 2 Department of Medicine and Science of Ageing, University G. D’Annunzio, Via Dei Vestini 31, 66100 Chieti, Italy; ludorotunno@gmail.com (L.R.); nelson.anzoletti@gmail.com (N.A.); m.zito@dmsi.unich.it (M.Z.)

**Keywords:** Alzheimer’s disease (AD), Electroencephalography (EEG), functional Near-Infrared Spectroscopy (fNIRS), multimodal neuroimaging, neurovascular coupling (NC), complexity analysis, sample entropy, conditional entropy, Rey–Osterrieth complex figure, Raven’s progressive matrices

## Abstract

Alzheimer’s disease (AD) is characterized by working memory (WM) failures that can be assessed at early stages through administering clinical tests. Ecological neuroimaging, such as Electroencephalography (EEG) and functional Near Infrared Spectroscopy (fNIRS), may be employed during these tests to support AD early diagnosis within clinical settings. Multimodal EEG-fNIRS could measure brain activity along with neurovascular coupling (NC) and detect their modifications associated with AD. Data analysis procedures based on signal complexity are suitable to estimate electrical and hemodynamic brain activity or their mutual information (NC) during non-structured experimental paradigms. In this study, sample entropy of whole-head EEG and frontal/prefrontal cortex fNIRS was evaluated to assess brain activity in early AD and healthy controls (HC) during WM tasks (i.e., Rey–Osterrieth complex figure and Raven’s progressive matrices). Moreover, conditional entropy between EEG and fNIRS was evaluated as indicative of NC. The findings demonstrated the capability of complexity analysis of multimodal EEG-fNIRS to detect WM decline in AD. Furthermore, a multivariate data-driven analysis, performed on these entropy metrics and based on the General Linear Model, allowed classifying AD and HC with an AUC up to 0.88. EEG-fNIRS may represent a powerful tool for the clinical evaluation of WM decline in early AD.

## 1. Introduction

Alzheimer’s disease (AD) is a form of dementia associated with memory failures that slowly decline into noticeable cognitive impairments [1]. AD is usually characterized by extracellular beta amyloid deposits [2], tau protein anomalies [3], neuronal loss [4], and neurovascular dysfunction [5,6]. However, since the physio-pathological mechanisms that produce AD symptoms are still not completely known [7], AD diagnosis is majorly performed through clinical tests that investigate the memory failures related to the dementia. Tests able to assess working memory (WM) impairments are often employed in clinical settings. For instance, the Rey–Osterrieth complex figure (ROCF) [8] is used to assess visuospatial functions and visuographic memory [9,10,11], and the standard Raven progressive matrices (RPM) [12] are widely used to assess visuospatial ability and abstract reasoning [13].

Indeed, the performance in a single test is not generally considered indicative of AD, and a large battery of tests is administered. In this perspective, neuroimaging could be employed during these tests in order to have a physiological correlate that could support AD diagnosis [6,14,15]. The main issue of this application is to maintain the ecological feature of the tests. In fact, a free interaction between the doctor and the patient is often required, and a neuroimaging technique such as functional magnetic resonance imaging (fMRI) is not suited to this aim. Conversely, neuroimaging techniques that provide less physical constrains such as Electroencephalography (EEG) and functional Near Infrared spectroscopy (fNIRS) are particularly suited.

EEG is a neuroimaging technique that is able to estimate the brain electrical activity measuring the electrical potential differences due to macroscopic currents within neuronal aggregates [16]. This technique is widely used in research and clinical settings to monitor brain function and to detect anomalies [17]. fNIRS is a non-invasive optical methodology that exploits the low absorption features of biological tissue in the near infrared spectral range to detect variations of the main absorbing chromophores in NIR, namely oxygenated (O_2_Hb) and deoxygenated (HHb) hemoglobin, in response to neuronal activity in the brain cortex [18]. This technique is portable, relatively cheap, lightweight, and resilient to motion artifacts [19], thus being suitable for ecological measurements during the administration of clinical tests. These two scalp-located techniques could be used concurrently, providing a multimodal neuroimaging tool that is able to measure the electrical and associated hemodynamic brain activity [20]. Multimodal EEG-fNIRS has been already utilized to assess cortical connectivity alterations associated with AD [21] and to perform a data-driven identification of AD, obtaining superior performances with respect to those obtained with standalone systems [22]. Notably, probing both the electrical and hemodynamic brain activity, it is possible to have information about the functional hyperemia in response to brain activity (i.e., neurovascular coupling, NC), which is known to be dysregulated in AD [23,24]. In fact, Hock et al. [25] found a reduced oxygenation and cerebral blood flow in AD in response to verbal fluency tasks produced by an impaired NC. This dysregulation is produced by depositions of amyloid-peptide in neuropil and vessels that could impair the hemodynamic regulation mechanism performed by neurons, glia, and vascular cells [26,27,28].

Another important issue related to the employment of neuroimaging instrumentation during the administration of clinical tests is related to data analysis. In fact, both EEG and fNIRS canonical data analysis requires a structured experimental paradigm, i.e., the start of the stimulation and its duration have to be known [29,30]. However, employing these methods in ambulatory settings is not feasible because of the need to preserve ecological features of the tests. Hence, novel statistical methods of analysis must be used. Particularly, it is known that the brain signal variability is indicative of its functioning. Specifically, this variability is generated from the interplay between single neurons and their neuronal circuits that allows the brain to self-organize itself in order to maximize the brain information capacity [31]. In turn, these findings explain the capacity of entropy of quantifying the brain’s information processing [32,33,34], given the direct correspondence between the variance and the amount of information. This approach revealed promising results in the assessment of altered state of consciousness, brain aging, and quantification of the brain networks’ information processing [31]. The complexity of cerebral signals can be evaluated using different entropy metrics. One such metric is the Sample Entropy (SampEn), which evaluates the non-linear predictability of a signal [35]. Moreover, always within the complexity metrics, it is also possible to evaluate the mutual complexity between two signals. The conditional entropy (CondEn) quantifies the amount of information needed to describe the outcome of a variable from the value of another variable [36]. Complexity evaluation is widely employed in neuroimaging to assess physiology and pathology [31]. In AD patients, it was utilized to analyze EEG signals acquired during resting state [37,38] and to investigate cortical activation during the execution of cognitive tasks employing fNIRS [39,40]. 

In the present study, the capability of complexity analysis to detect WM impairments in early AD with respect to healthy controls (HC) was investigated. SampEn from whole-head EEG and frontal/prefrontal cortex fNIRS signals concurrently acquired during two WM tasks was evaluated. Moreover, in order to have information about NC dysregulation in AD during the execution of WM tasks, CondEn between EEG and fNIRS was also computed. In detail, whole-head EEG power envelopes in five frequency bands (theta [θ], alpha [α], beta [β], delta [δ], and gamma [γ]) and frontal changes of O_2_Hb and HHb were considered, resulting in 3 EEG SampEn metrics, 2 fNIRS SampEn metrics and 10 NC CondEn metrics. The coupling between electrical and hemodynamic brain activity was evaluated convolving the EEG signal with the canonical hemodynamic response. Finally, a cross-validated multivariate data-driven (i.e., Machine Learning) analysis based on a General Linear Model [41,42,43] employing all the evaluated complexity metrics as input was performed to classify AD and HC. Notably, the multivariate approach proposed provided a single dependent variable (i.e., label of the disease) and multiple independent features (i.e., complexity metrics). This framework was built to demonstrate the robustness of the findings and to provide an approach useful in clinical settings to support AD diagnosis.

## 2. Materials and Methods 

### 2.1. Participants

Thirty-five participants were enrolled in the study. The study sample was composed of 17 AD patients (mean age: 67.6 years; standard deviation (SD): 9.3 years; 9 females) and 18 HC (mean age: 69.2 years; SD: 9.1 years; 9 females). All the AD patients enrolled had a diagnosis of mild probable Alzheimer’s disease, as defined by the Diagnostic and Statistical Manual of Mental Disorders, 5th edition (DSM-5). The exclusion criteria were moderate to severe cognitive impairment (Mini Mental State Examination, MMSE < 25/30) [44], vascular dementia, behavioral or psychiatric disorders, brain lesions, history of stroke, and traumatic brain injury. The research was approved by the Research Ethics Board of the University G. D’Annunzio of Chieti-Pescara, Italy (approval number: 1479, date of approval: 03/05/2017), and it was performed in accordance to the principles of the Declaration of Helsinki. Informed consent was signed by all the participants before the experiment, and they could withdraw from it at any time.

### 2.2. Experimental Design

**Figure 1:**
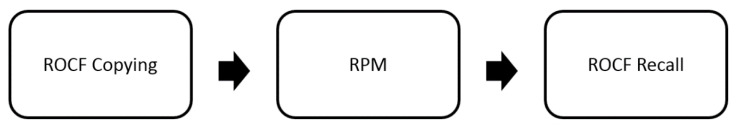
Experimental paradigm. The tasks were consecutively administered to the participants and separated by 1-min rest periods, as they are usually performed in clinical practice.

ROCF and RPM were administered by the doctor, as they are usually performed in clinical practice, preserving the free interaction and the ecological features of the tests. ROCF is composed of two phases: in the first phase, the patient is requested to reproduce a complex two-dimensional image (copying) whereas, in the second phase, the subject must draw the image again from memory (recall). The two phases are separated by a period of 10 min. During this period, an RPM test was administered. RPM consisted of filling empty spaces, choosing among four alternatives following a logical hunch. It is composed of five sets of items that follow different logic rules and become progressively more difficult during the set. Between ROCF phases and RPM, 1 min of rest was provided in order to remove eventual confounding cross-effects between the tests. The experimental paradigm is described in Figure 1.

### 2.3. Electroencephalograpy Instrumentation

**Figure 2:**
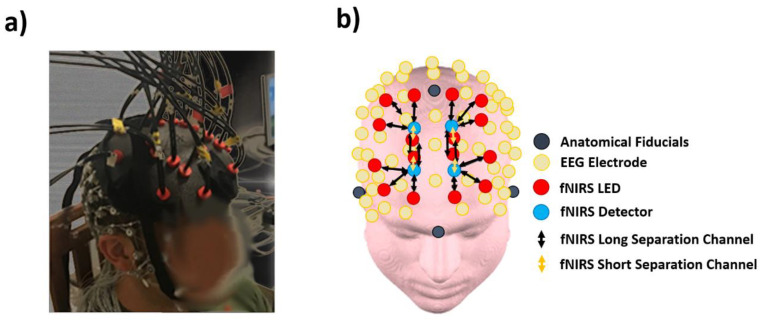
(**a**) Electroencephalography (EEG)-fNIRS (functional Near Infrared Spectroscopy) probes placed onto an indicative participant head. The fNIRS optodes were in contact with the scalp exploiting the space among the electrodes of the EEG cap. (**b**) EEG-fNIRS probes projected onto a template head. The high-density EEG layout was in agreement with the 10/20 system, and the fNIRS probes were positioned with reference to the EEG electrodes.

A high-density, 128 channel, full-head EEG instrumentation (Electrical Geodesic Inc, Eugene, OR, USA, EEG System Net 300, Figure 2a) was employed in the study to collect brain electrical activity. The impedance between scalp and electrodes was checked before each recording and values below 50 kΩ were considered acceptable. It is worth underlining that although a skin/sensor impedance below 5 kΩ is generally necessary for reliable EEG recordings, the HydroCel Geodesics Sensor Net succeed in measuring high-quality signals with impedances up to 50–100 kΩ thanks to the high-input impedance amplifiers [45]. The sample frequency was set at 250 Hz.

### 2.4. Functional Infrared Sprectroscopy Instrumentation

A frequency-domain NIRS system (Imagent, ISS Inc., Champaign, IL, USA) was used for the optical measurements. The system provided 32 laser diodes sources (16 emitting at 690 nm of wavelength and 16 emitting at 830 nm of wavelength) and 4 photomultiplier-tube (PMT) detectors. The sources were time-multiplexed in order to prevent their crosstalk. The sampling rate was 10.42 Hz. Sources and detectors were located on the frontal and prefrontal cortices through a home-made optical patch located on top of the high-density EEG cap (Figure 2a). Notably, the optodes were placed in contact with the scalp exploiting the space among the electrodes of the EEG cap, allowing also placing the optical array with reference to the 10/20 system [46] (Figure 2b). The optical array allowed collecting optical data from 16 long separation channels at source-detector distances of 35 mm and from four short separation channels at 15 mm interoptode distance (Figure 2b). The short separation channels are sensitive to hemoglobin concentration changes in the scalp; hence, they allow correcting the long separation channels (which are sensitive both to extracranial and intracranial hemoglobin oscillations) for superficial hemoglobin variations [47,48,49].

### 2.5. Electroencephalograpy Signal Pre-Processing

Firstly, EEG signals were visually inspected to reject saturated or corrupted epochs. A band-pass filter (cut-off frequencies: 1 and 80 Hz) and a notch-filter at 50 Hz were applied (zero-lag 2nd order Butterworth digital filters). Furthermore, a procedure relying on Independent Component Analysis (ICA) was applied to remove cardiac, ocular, and muscular artifacts [50,51]. The pre-processed EEG signals were decomposed in five frequency bands of interest (θ-band: 3.5–8.2 Hz, α-band: 7.4–13 Hz, β-band: 13–30 Hz, δ-band: 1–4 Hz, γ-band: 26–40 Hz), and the power temporal envelopes were evaluated as the absolute values of their Hilbert transform.

### 2.6. Functional Near Infrared Spectroscopy Pre-Processing

The raw continuous-wave component of the fNIRS signal was converted into optical densities (ODs) according to the equation:

OD = −ln(I(t)/I_avg_) (1)

where I(t) is the signal intensity over time and I_avg_ is its average value. Then, motion artifacts were removed by means of a wavelet-based procedure [52] and the *OD*s were band-pass filtered with a zero-lag, 4th order Butterworth digital filter (cut-off frequencies of 0.01 Hz and 0.4 Hz). Oscillations in the concentration of *O*_2_*Hb* and *HHb* were computed for each channel employing the modified Lambert–Beer Law [53]:

(2) $$\left(\frac{\Delta O_{2}Hb}{\Delta HHb}\right)=\frac{1}{d}{\left[\begin{array}{cc} \varepsilon_{O_{2}Hb}\left(\lambda_{1}\right)DPF\left(\lambda_{1}\right) & \varepsilon_{HHb}\left(\lambda_{1}\right)DPF\left(\lambda_{1}\right)\\ \varepsilon_{O_{2}Hb}\left(\lambda_{2}\right)DPF\left(\lambda_{2}\right) & \varepsilon_{HHb}\left(\lambda_{2}\right)DPF\left(\lambda_{2}\right) \end{array}\right]}^{-1}\times \;\left[\begin{array}{c} \Delta OD\left(\lambda_{1}\right)\\ \Delta OD\left(\lambda_{2}\right) \end{array}\right]$$

where *d* is the geometrical interoptode distance, *ε* is the extinction coefficient for the specific chromophore at a given wavelength (*λ*), and DPF is the Differential Pathlength Factor. Particularly, an accurate evaluation of the *DPF* is fundamental to reduce the crosstalk between the two haemoglobin forms; hence, in this study, it was computed accordingly to [54,55]. The short separation channels were utilized to remove the extracranial hemodynamic contribution in the long separation channels [48]. Particularly, short channels were employed to remove the scalp confoundings from the long separation channels in accordance with [56]. This method relies on GLM and Principal Component Analysis (PCA). Specifically, the first principal component of the short channels is used to define a global scalp-hemodynamic model, which is used as a regressor of the GLM to assess its influence over the long separation channels. Thus, it is possible to eliminate the global scalp-hemodynamic confounding from each long separation channel signal by subtracting the global scalp-hemodynamic model multiplied by the β-values associated to the global scalp-hemodynamic model for a specific channel [56].

### 2.7. Complexity Analysis

SampEn is defined as the negative natural logarithm of the conditional probability that signal subseries of length m (pattern length) that match pointwise within a tolerance r (similarity factor) also match at the m + 1 point. SampEn was evaluated for the global field potential (GFP) [57] of the EEG power temporal envelopes in the five frequency bands of interest (i.e., α-band, θ-band, β-band, δ-band, and γ-band) and for the two hemoglobin forms (i.e., O_2_Hb and HHb) computed from average fNIRS signals across all the measurement channels during each experimental phase. Notably, for the computation of the average fNIRS signal, only the long separation channels were employed.

SampEn of a time series {x_1_,…,x_N_} of length N is computed employing the following set of equations [58]:

(3) $$\mathrm{SampEn}\left(m,r,N\right)=-\ln\left[\frac{U^{m+1}\left(r\right)}{U^{m}\left(r\right)}\right]$$

$$U^{m}\left(r\right)={\left[N-m\tau \right]}^{-1}\overset{N-m\tau }{\underset{i=1}{\sum }}C_{i}^{m}\left(r\right)$$

$$C_{i}^{m}\left(r\right)=\frac{B_{i}}{N-\left(m+1\right)\tau }.$$

Essentially, the functions $C_{i}^{m}\left(r\right)$ are conditional probabilities calculated as a sum of the (matches)/(total of possible vectors) among all the target vectors. The parameters of these functions are described below:

$$B_{i}=\mathrm{number}\;\mathrm{of}\;j\;\mathrm{where}\;d\left|X_{i},X_{j}\right|\leq r$$

$$X_{i}=\left(x_{i},x_{i+\tau }\ldots ,x_{i+\left(m-1\right)\tau }\right)$$

$$X_{j}=\left(x_{j},x_{j+\tau }\ldots ,x_{j+\left(m-1\right)\tau }\right)$$

i≤j≤N−mτ, j≠i

where N is the total length of the time-series considered, m is the embedded dimension, r is the tolerance factor (scalar for which two subseries with distances below its value are considered identical), and τ is the time delay expressed in samples. In this study, the embedded dimension was m = 2 and the similarity factor r = 0.2 × SD of the signal. These parameters are commonly employed for complexity analysis of biological signals and they were chosen in accordance with [35]. SampEn was evaluated using the following software: Víctor Martínez-Cagigal (2018). Sample Entropy. Mathworks.

CondEn is indicative of the information needed to describe the outcome of a random variable given the value of another random variable, and it could be evaluated as follows:

(4) $$H\left(Y|X\right)=-\underset{x\in X}{\sum }\underset{y\in Y}{\sum }p\left(x,y\right)\log p\left(y|x\right)$$

where ***x*** and ***y*** denote the support sets of ***X*** and ***Y***, while ***p*** (***x***, ***y***) and ***p*** (***y***|***x***) are the values of their joint and conditional probability distributions. Similar to SampEn, CondEn was evaluated on the GFP of the EEG channels and the average of fNIRS signals across all the channels (only fNIRS long separation channels were considered). In order to take into account the different temporal scale of the EEG and fNIRS signals, the EEG signal was convolved with the canonical hemodynamic response [59] and then down-sampled to the sample frequency of the fNIRS signal [60]. CondEn was evaluated by means of the follow software package: Information Theory Toolbox (https://www.mathworks.com/matlabcentral/fileexchange/35625-information-theory-toolbox, Mo Chen, 2020). 

Importantly, given the ecological feature of the experimental paradigm, the temporal length of the different phases across subjects was different. Since the evaluation of the complexity metrics could be sensitive to the duration of the signal, for the evaluation of the metrics, the epochs associated to the different experimental phases were cut at the same duration of the one which lasts less (around 4 min).

Notably, previously to evluate SampEn and CondEn, the stationarity of the EEG and fNIRS time series was checked employing the Phillips–Perron test, and, if the signals were not stationary, a detrending was applied. The complexity metrics were computed for further analysis only for the stationary time series.

### 2.8. Statistical Inference and Multivariate Classification

The 95% confidence interval (95% C.I.) of SampEn and CondEn was evaluated by a bootstrap procedure. Only the values within the confidence intervals were used for further statistical analysis.

Unpaired *t*-tests were employed to compare the complexity metrics evaluated from AD with HC. False Discovery Rate (FDR) correction for multiple comparisons was employed. Furthermore, a data-driven multivariate analysis based on GLM was implemented to provide a classification of disease (AD or HC). Three linear regressions were evaluated employing separately the complexity metrics evaluated from the unimodal and multimodal recordings (i.e., 5 EEG SampEn, 2 fNIRS SampEn, 10 NC CondEn) and the dependent variable labeled the presence of the disease (AD = 1, HC = 0). In order to provide the generalization performances of the classifier, a leave one out cross-validation procedure was implemented. A Receiver Operating Characteristic (ROC) curve analysis on the out-of-sample predicted outputs was performed to provide an estimation of the sensitivity and specificity to the disease of the complexity metrics in each experimental phase. Importantly, the classifiers were fed employing all the features evaluated, independently from the descriptive statistic results.

## 3. Results

Table 1 reports the values of the EEG, fNIRS, and neurovascular coupling (NC) complexity metrics (mean value ± SD) and associated 95% C.I. evaluated during the different experimental phases.

Table 2 reports the results of the *t*-test between AD and HC regarding the EEG, fNIRS, and NC complexity metrics evaluated during the different experimental phases.

**Figure 3:**
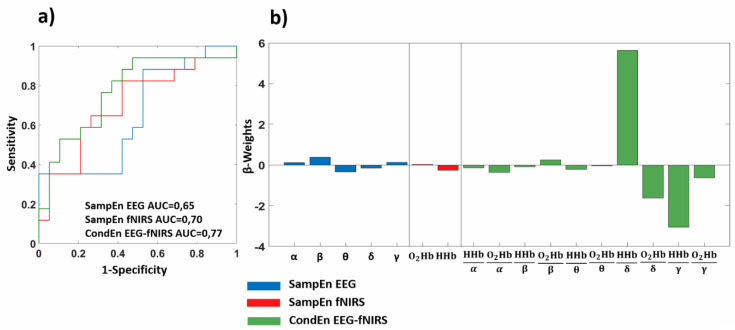
Classification results related to Rey–Osterrieth complex figure (ROCF) (copying). (**a**) Receiver Operating Characteristic (ROC) curve obtained employing the cross-validated classification performed using all the complexity metrics evaluated; (**b**) General Linear Model (GLM) β-weights associated to each regressor.

Figure 3 reports the results of the machine learning approach related to ROCF (copying). Figure 3a reports the ROC curve associated to the leave-one-out cross-validated GLM-based classification performed using as input the different complexity metrics evaluated, whereas Figure 3b reports the β-weights associated to each regressor. Concerning the SampEn of the EEG signal, an Area Under the Curve (AUC) of 0.65 was obtained. Choosing a threshold of 0.64 on the output of the GLM machine learning framework, a sensitivity of 0.88 and a specificity of 0.47 were achieved. Regarding the fNIRS complexity metrics, the procedure delivered an AUC of 0.70, and setting a threshold of 0.53 on the cross-validated output, a sensitivity of 0.65 and a specificity of 0.74 were achieved. With respect to the multimodal EEG-fNIRS metrics, an AUC of 0.77 was delivered, and choosing a threshold of 0.42 of the cross-validated output, a sensitivity of 0.76 and a specificity of 0.68 were reached.

**Figure 4:**
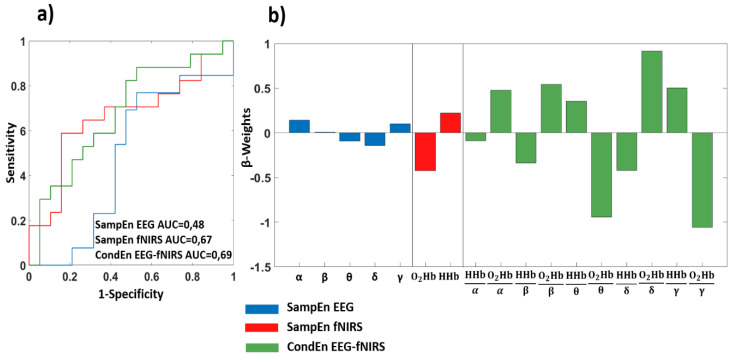
Classification results related to Raven progressive matrices (RPM). (**a**) ROC curve obtained employing the cross-validated classification performed using all the complexity metrics evaluated; (**b**) GLM β-weights associated to each regressor.

Figure 4 reports the results of the data-driven procedure applied to RPM. Figure 4a reports the ROC curve associated to the leave-one-out cross-validated output of the machine learning framework, whereas Figure 4b shows the β-weights associated to each regressor. Using the SampEn EEG metrics, an AUC of 0.48 was obtained. Employing the fNIRS complexity metrics, an AUC of 0.67 was delivered, and selecting a threshold of 0.54 on output of the multivariate analysis, a sensitivity of 0.65 and a specificity of 0.74 were obtained. The ROC curve associated to the CondEn EEG-fNIRS exhibited an AUC of 0.69, and using a threshold of 0.42, a sensitivity of 0.71 and a specificity of 0.58 were reached. 

**Figure 5:**
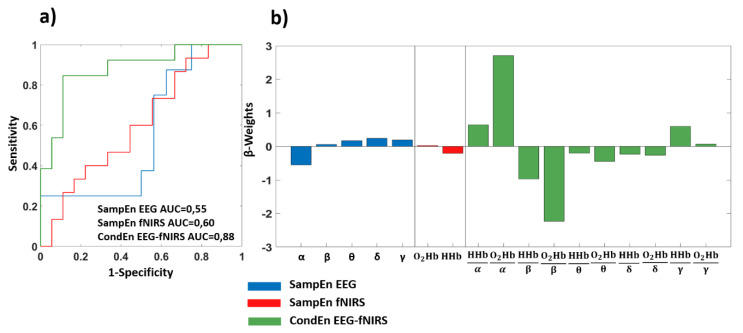
Classification results related to ROCF (recall). (**a**) ROC curve obtained employing the cross-validated classification performed using all the complexity metrics evaluated; (**b**) GLM β-weights associated to each regressor.

Figure 5 shows the results of the machine learning framework associated to the ROCF (recall). Figure 5a reports the ROC curve associated to the leave-one-out cross-validated classification and Figure 5b represents the β-weights relative to each regressor. Concerning the EEG results, an AUC of 0.55 was delivered, and setting a threshold of 0.66 on the cross-validated output, a sensitivity of 0.75 and a specificity of 0.44 were reached. Regarding the fNIRS SampEn, an AUC of 0.60 was obtained, and using a threshold of 0.45 on the output, a sensitivity of 0.60 and a specificity of 0.66 were delivered. Employing the CondEn EEG-fNIRS complexity metrics, the data-driven procedure delivered an AUC of 0.88, and setting a threshold of 0.56 on the output, a sensitivity of 0.85 and a specificity of 0.89 were reached. 

Comparing the performances of the three data-driven procedures implemented during the different experimental phases, the multimodal EEG-fNIRS NC metrics delivered a statistically significant higher AUC with respect to unimodal EEG and fNIRS during the ROCF (recall) (CondEn EEG-fNIRS vs. SampEn EEG: z-stat = 1.977; *p* = 0.048; CondEn EEG-fNIRS vs. SampEn fNIRS: z-stat = 2.955; *p* = 0.003).

## 4. Discussion

The aim of this study was to assess the feasibility of employing ecological and multimodal EEG-fNIRS neuroimaging during clinical tests that investigate WM abilities (i.e., ROCF and RPM). To preserve the ecological features of these cognitive tests and to maintain a free interaction between the doctor and patients, brain activity was estimated employing a complexity metrics, which does not require a structured paradigm. Specifically, in this study, SampEn was employed to estimate the electrical and hemodynamic brain activity. Moreover, since synchronous EEG and fNIRS measurements allow evaluating the NC, the mutual information between the two signals was estimated through the CondEn. Notably, CondEn measures the quantity of entropy a variable has remaining once the value of a second variable is known. Hence, it evaluated the remaining of entropy of the hemodynamic signal when the electrical signal was known, thus describing their interaction, and, consequently, the NC, which is known to be dysregulated in AD. It is worth noting that the dependence of the hemodynamic signal from the electrical signal could have been evaluated employing different metrics with respect to CondEn (e.g., covariance and cross-correlation). However, complexity metrics such as CondEn and SampEn are able to estimate the predictability of the signals, which could be indicative of altered brain activations [31]. Indeed, complexity metrics are able to quantify the amount of information of brain signals, which could be more suggestive of pathologies with respect to the simple variability.

The results showed statistically significant differences in both electrical and hemodynamic brain activities between the two groups (i.e., AD and HC). Specifically, the descriptive statistics employed highlighted differences during all the experimental phases between AD and HC for almost all the global EEG, fNIRS, and NC metrics. During ROCF (Copying), the SampEn of the two hemoglobin forms and CondEn of the NC metrics associated to δ- and γ-bands were higher in AD with respect to HC. Concerning RPM, only O_2_Hb/β-band appeared to be lower in AD with respect to HC after FDR correction. Regarding ROCF (copying), almost all NC metrics were significantly higher in HC with respect to AD. In previous study, it was demonstrated that lower values of complexity are associated to brain activations; hence, it is licit to suppose that HC exhibited a lower brain activation with respect to AD during the execution of WM tasks. These results are in line with previous studies that employed complexity metrics to evaluate hemodynamic brain activity in AD [39,40], depicting a lower brain activation in HC. Concerning the EEG results, it was demonstrated that AD patients exhibit a lower SampEn of EEG signal with respect to HC during the resting state [37]; moreover, as reported by De Bock et al., the ratio of Tsallis entropy evaluated over frontal and occipital/temporal cortices during WM tasks is indicative of AD [61]. However, the approach of the present study and the one proposed by De Bock are quite different; thus, it is difficult to perform a comparison. Nonetheless, it supports the hypothesis that the complexity of EEG signal during WM tasks could be indicative of the cognitive decline in AD. Moreover, it was demonstrated that a θ-band activity is associated to WM tasks [62], confirming the strong effect on this frequency band found in this study. Regarding NC results, it was demonstrated that the remaining entropy of the hemodynamic signal, when the EEG signal is known, is higher in HC with respect to AD. To the best of our knowledge, studies evaluating NC employing synchronous EEG-fNIRS in AD are missing; however, some studies using EEG-fMRI are available. Specifically, a previous work investigated the correlations between EEG and the fMRI blood oxygen level dependent (BOLD) effect on healthy participants during WM tasks [63]. They demonstrated that EEG-BOLD signal correlations changes across the different brain regions and EEG frequency bands, and the load analysis showed that θ-, β-, and γ-bands had exclusively positive load effects, confirming the involvement of these bands in this kind of task, as reported in this study.

In order to demonstrate the robustness of the findings, a data-driven machine learning approach based on GLM was implemented. The output of the classification was defined in accordance with the diagnosis received by the patients (HC = 0; AD = 1). The results confirmed that EEG, fNIRS, and NC complexity metrics could discriminate the two populations during the execution of almost all the experimental phases. Specifically, EEG metrics seemed to have lower abilities to discriminate HC and AD with respect to the other metrics. It is worth underlining that NC metrics exhibited a statistically higher capability of classifying the disease during the ROCF (recall) with respect to both fNIRS and EEG. Moreover, although not significantly, NC metrics exhibited always higher performances in classifying the two groups with respect to the unimodal recordings, demonstrating the importance of employing a simultaneous EEG-fNIRS system in clinical settings. Importantly, an ROC curve shows the variation of the sensitivity and specificity of a test as a function of the variable of interest. Hence, by setting a threshold of this variable, it is possible to obtain different values of sensitivity and specificity. Generally, the threshold is chosen in accordance with the aim of the application (e.g., a great specificity is needed, and a low specificity is acceptable). The values reported in this study were chosen in order to obtain a good compromise between sensitivity and specificity, but it could be possible to consider different values. 

Importantly, the EEG, fNIRS, and NC features were used as input of three different classifiers in order to test the capability of the single unimodal approach (i.e., EEG and fNIRS) and of the multimodal technique (i.e., NC evaluated as CondEn) to discriminate the presence of the disease. It was not possible to employ all the features together (i.e., 5 EEG SampEn, 2 fNIRS SampEn, 10 NC CondEn = 17 features) because the number of the features is equal to the subjects of the AD class, thus possibly introducing an overfitting effect to the classification.

A linear model allows evaluating the contribution of the single features to the estimation of the output. Concerning EEG metrics, the highest GLM β-value is associated to SampEn of the β-band for ROCF (copying), whereas SampEn of the α-band is the regressor that most contributes to the classification of the pathology during RPM and ROCF (recall). Concerning fNIRS complexity metrics, SampEn of HHb was the regressor with the highest contribution during ROCF (copying) and ROCF (recall), whereas O_2_Hb majorly contributed to the estimation of the pathology during RPM. Regarding the NC metrics, HHb/δ-band exhibited the highest β-value during ROCF (copying), O_2_Hb/γ-band showed the highest value during RPM, and O_2_Hb/α-band majorly contributed to the discrimination of the two groups during ROCF (recall). 

These results are in line with previous works performed on HC. In fact, a strong negative correlation of the α-band with BOLD acquired over parietal and frontal cortex was found [64], whereas a positive relation was revealed at rest between BOLD and the θ-band of Local Field Potentials in parahippocampal areas [65]. Thus, the amplitude and the sign of the β-weights associated to the α-band and θ-band could simply reflect a global neurovascular uncoupling accompanying the disease that become more evident for those frequency bands and hemoglobin forms where the original physiological interaction is predominant. Moreover, an increase in δ-band power during mental tasks has been already observed in the literature, and it is associated with functional cortical deafferentation or inhibition of the sensory afferences that obstruct the internal concentration [66].

These findings suggest a possible relevance of neuroimaging tools, such as multimodal EEG-fNIRS, in clinical practice to support early AD diagnosis. These technologies could be easily employed in the outpatient environment since they are relatively cheap, portable, and easy to use; hence, they do not require specialized operators. Furthermore, employing a complexity analysis allows preserving the ecological feature of the tests and the free doctor–patients interaction. In addition, the results of this study are relative to a global whole head EEG and frontal/prefrontal fNIRS metrics. It should be stressed that employing an average index of complexity is useful in clinical applications where a perfect co-registration between the neuroimaging sensors and the anatomical structures of the patients is not feasible. Particularly, in order to perform a correct co-registration, it is necessary to obtain a structural MRI of the patients, making this approach expensive and quite unfeasible in routine clinical practice. 

One limitation of this study was to employ a whole-head EEG system and an fNIRS device that covers only the frontal and prefrontal cortices. This limitation is due to the limited number of optodes of the fNIRS system available that did not allow covering the whole scalp. Hence, it was preferred to cover the frontal and prefrontal cortex, since these areas are involved in WM tasks [67].

However, further studies should be performed, increasing the population sample size, which might improve the multivariate complexity-based classification outcome. Notably, the classification was conducted employing a leave-one-out cross-validation procedure (i.e., removing one subject at a time and testing the classifier on that specific subject), thus intrinsically evaluating the out-of-sample performance of the classifier, making the results obtained generalizable. However, increasing the sample size may allow further improvement of the performance by decreasing a possible in-sample overfitting effect of the classifier. Furthermore, enrolling more participants could allow employing all the complexity metrics evaluated in this study as input of the proposed GLM-based classifier without incurring in overfitting issues. 

Moreover, it could be worth employing more advanced classification procedures (e.g., Deep Learning [68]), which were not usable in this work given the small sample size and the possible over-fitting effect. Finally, it could be interesting to further investigate the importance of the relationship and interaction between the physician and the patients, for instance implementing hyperscanning procedures [69]. 

Indeed, this study did not provide an alternative tool for early AD diagnosis, but it could pave the way to the introduction of synchronous EEG-fNIRS technologies to support clinical procedures aimed at investigating cognitive decline associated to dementia.

## 5. Conclusions

In this study, the capability of multimodal EEG-fNIRS together with complexity analysis (i.e., SampEn and CondEn) to classify early AD and HC during tests that assess WM abilities (ROCF and RPM) was investigated. The global SampEn of five EEG bands (i.e., α-band, β-band, θ-band, δ-band, and γ-band) and two hemoglobin fNIRS signals (i.e., O_2_Hb and HHb), as well as the CondEn between the five EEG bands and the two fNIRS hemoglobin signals (i.e., O_2_Hb/α, HHb/α, O_2_Hb/β, HHb/β, O_2_Hb/θ, HHb/θ, O_2_Hb/δ, HHb/δ, O_2_Hb/γ, and HHb/γ, depicting the NC) demonstrated the effectiveness of the approach to discriminate AD and HC during the execution of WM tasks. A multivariate analysis of the complexity metrics evaluated based on the general linear model provided a good classification of the disease. These results, although preliminary, seem to confirm the hypothesis that AD may produce a dysregulation of brain electrical activity and neurovascular coupling that may be exploited in clinical practice to support early AD diagnosis.

## Figures and Tables

**Table 1:** Mean values and associated SD of the EEG, fNIRS, and NC complexity metrics and relative 95% C.I. evaluated during the different experimental phases for the AD and HC groups.

	Metric	AD (Mean ± SD)	HC (Mean ± SD)	AD (95% C.I.)	HC (95% C.I.)
	α-band	0.357 ± 0.059	0.318 ± 0.071	0.194–0.500	0.026–0.540
	β-band	0.361 ± 0.059	0.265 ± 0.128	0.186–0.512	−0.013–0.540
	θ-band	0.277 ± 0.075	0.251 ± 0.118	0.117–0.438	−0.001–0.503
	δ-band	0.914 ± 0.348	0.909 ± 0.224	0.077–1.817	0.424–1.387
	γ-band	0.899 ± 0.316	0.923 ± 0.282	0.059–1.644	0.030–1.776
	O_2_Hb	0.121 ± 0.037	0.170 ± 0.035	0.032–0.219	0.081–0.249
	HHb	0.129 ± 0.043	0.170 ± 0.038	0.038–0.221	0.088–0.253
ROCF	O_2_Hb/α-band	1.306 ± 0.301	1.585 ± 0.261	0.648–1.971	0.939–2.316
(Copying)	HHb/α-band	0.040 ± 0.027	0.053 ± 0.041	−0.032–0.128	−0.105–0.248
	O_2_Hb/β-band	1.249 ± 0.325	1.687 ± 0.436	0.549–1.941	0.744–2.615
	HHb/β-band	0.046 ± 0.033	0.057 ± 0.049	−0.037–0.141	−0.108–0.254
	O_2_Hb/θ-band	3.985 ± 0.701	4.258 ± 0.787	2.314–5.879	2.349–6.494
	HHb/θ-band	0.082 ± 0.040	0.085 ± 0.062	−0.019–0.197	−0.152–0.370
	O_2_Hb/δ-band	3.952 ± 0.455	3.387 ± 0.573	1.558–5.138	1.969–5.017
	HHb/δ-band	3.963 ± 0.465	3.401 ± 0.581	1.573–5.156	1.929–5.106
	O_2_Hb/γ-band	3.866 ± 0.441	3.324 ± 0.569	1.464–5.053	1.975–5.880
	HHb/γ-band	3.939 ± 0.470	3.381 ± 0.577	1.574–5.143	1.954–5.020
	α-band	0.394 ± 0.033	0.382 ± 0.048	0.320–0.468	0.279–0.485
	β-band	0.378 ± 0.044	0.386 ± 0.054	0.237–0.497	0.242–0.512
	θ-band	0.338 ± 0.085	0.371 ± 0.060	0.150–0.527	0.204–0.517
	δ-band	1.796 ± 0.322	1.897 ± 0.152	1.081–2.513	1.484–2.259
	γ-band	1.758 ± 0.288	1.789 ± 0.342	1.099–2.412	0.784–2.660
RPM	O_2_Hb	0.171 ± 0.049	0.209 ± 0.040	0.066–0.277	0.102–0.332
	HHb	0.180 ± 0.047	0.211 ± 0.046	0.079–0.282	0.096–0.341
	O_2_Hb/α-band	3.454 ± 0.728	3.928 ± 0.241	1.872–5.036	3.339–4.599
	HHb/α-band	2.501 ± 1.082	3.148 ± 0.605	0.107–4.860	1.827–4.456
	O_2_Hb/β-band	3.351 ± 0.656	4.025 ± 0.449	1.916–4.786	3.041–4.996
	HHb/β-band	2.438 ± 1.039	3.191 ± 0.711	0.204–4.689	1.703–4.714
	O_2_Hb/θ-band	3.357 ± 0.705	3.939 ± 0.324	1.847–4.891	3.184–4.787
	HHb/θ-band	2.433 ± 1.055	3.181 ± 0.653	0.132–4.735	1.792–4.577
	O_2_Hb/δ-band	3.344 ± 1.455	3.906 ± 0.575	0.771–6.401	0.488–5.547
	HHb/δ-band	4.691 ± 1.416	4.654 ± 0.645	1.623–6.716	1.264–6.057
	O_2_Hb/γ-band	3.951 ± 1.264	3.996 ± 0.777	0.924–5.429	0.833–5.518
	HHb/γ-band	5.068 ± 1.351	5.554 ± 0.591	2.077–7.055	2.061–7.281
	α-band	1.675 ± 0.418	1.800 ± 0.295	0.625–2.735	1.160–2.437
	β-band	1.467 ± 0.578	1.731 ± 0.344	0.007–2.936	0.651–2.661
	θ-band	1.679 ± 0.219	1.562 ± 0.442	1.133–2.221	0.604–2.520
	δ-band	1.956 ± 0.174	1.877 ± 0.233	0.580–3.003	1.180–2.484
	γ-band	1.775 ± 0.262	1.580 ± 0.545	1.100–2.452	0.340–2.761
ROCF	O_2_Hb	0.114 ± 0.036	0.138 ± 0.039	0.033–0.193	0.039–0.252
(Recall)	HHb	0.112 ± 0.037	0.146 ± 0.049	0.032–0.192	0.040–0.251
	O_2_Hb/α-band	0.662 ± 0.260	1.299 ± 0.402	0.075–1.256	0.302–2.463
	HHb/α-band	0.312 ± 0.226	0.528 ± 0.222	−0.430–1.208	−0.015–1.163
	O_2_Hb/β-band	0.665 ± 0.258	1.298 ± 0.410	0.087–1.249	0.293–2.453
	HHb/β-band	0.310 ± 0.222	0.531 ± 0.225	−0.456–1.250	−0.013–1.155
	O_2_Hb/θ-band	0.660 ± 0.256	1.295 ± 0.406	0.081–1.234	0.278–2.452
	HHb/θ-band	0.312 ± 0.227	0.792 ± 0.130	−0.438–1.236	0.467–1.161
	O_2_Hb/δ-band	2.488 ± 0.541	3.456 ± 0.522	1.258–3.708	2.345–4.582
	HHb/δ-band	1.682 ± 0.876	2.276 ±0.659	−0.277–3.693	0.834–3.707
	O_2_Hb/γ-band	2.737 ± 0.654	3.987 ± 0.787	1.299–4.184	2.287–5.689
	HHb/γ-band	1.819 ± 0.816	2.486 ± 0.737	−0.466–4.098	0.898–4.103

**Table 2:** *t*-test results of the complexity metrics evaluated during the different experimental phases (* *p* < 0.05, False Discovery Rate (FDR) corrected).

	Metric	T-Stat	D.f.	*p*-Value	Effect Size (D-Cohen)
	α-band	1.697	31	0.0997	0.590
	β-band	2.743	33	0.010	0.940
	θ-band	0.766	33	0.449	0.256
	δ-band	0.056	33	0.956	0.019
	γ-band	−0.304	32	0.763	−0.104
	O_2_Hb	−4.082	32	3 × 10^−4^ *	−1.4025
	HHb	−3.026	33	0.0047 *	−1.0102
ROCF	O_2_Hb/α-band	−2.891	31	0.007 *	−0.980
(Copying)	HHb/α-band	−1.040	31	0.306	−0.358
	O_2_Hb/β-band	−3.380	32	0.002 *	−1.129
	HHb/β-band	−0.698	33	0.490	−0.240
	O_2_Hb/θ-band	−1.059	32	0.297	−0.364
	HHb/θ-band	−0.162	32	0.872	−0.056
	O_2_Hb/δ-band	3.156	32	0.004 *	1.085
	HHb/δ-band	3.085	32	0.004 *	1.060
	O_2_Hb/γ-band	3.079	32	0.004 *	1.058
	HHb/γ-band	3.067	32	0.004 *	1.054
	α-band	0.812	31	0.423	0.292
	β-band	−0.415	33	0.681	−0.155
	θ-band	−1.271	32	0.214	−0.465
	δ-band	−1.175	33	0.250	−0.432
	γ-band	−0.261	32	0.796	−0.095
RPM	O_2_Hb	−2.498	33	0.018	−0.847
	HHb	−1.956	33	0.059	−0.662
	O_2_Hb/α-band	−2.614	31	0.014	−0.909
	HHb/α-band	−2.232	31	0.033	−0.757
	O_2_Hb/β-band	−3.597	33	0.001 *	−1.221
	HHb/β-band	−2.537	33	0.016	−0.861
	O_2_Hb/θ-band	−3.151	32	0.004	−1.093
	HHb/θ-band	−2.566	32	0.015	−0.871
	O_2_Hb/δ-band	1.172	31	0.250	0.409
	HHb/δ-band	0.104	33	0.918	0.035
	O_2_Hb/γ-band	−0.126	31	0.901	−0.044
	HHb/γ-band	−1.386	32	0.175	−0.481
	α-band	−0.849	32	0.405	−0.367
	β-band	−1.381	29	0.182	−0.610
	θ-band	0.706	30	0.488	0.306
	δ-band	0.790	31	0.439	0.363
	γ-band	0.951	31	0.352	0.412
ROCF	O2Hb	−1.787	30	0.084	0.633
(Recall)	HHb	−2.204	31	0.035	−0.771
	O_2_Hb/α-band	−4.961	31	3.088 × 10^−5^ *	−1.817
	HHb/α-band	−2.560	30	0.016	−0.965
	O_2_Hb/β-band	−4.859	28	4.080 × 10^−5^ *	−1.779
	HHb/β-band	−2.618	27	0.014	−0.987
	O_2_Hb/θ-band	−4.923	30	3.420 × 10^−5^ *	−1.803
	HHb/θ-band	−7.233	29	8.862 × 10^−8^ *	−2.717
	O_2_Hb/δ-band	−5.021	30	2.396 × 10^−5^ *	−1.827
	HHb/δ-band	−2.160	30	0.039	−0.786
	O_2_Hb/γ-band	−4.672	31	6.313 × 10^−5^ *	−1.700
	HHb/γ-band	−2.124	30	0.042	−0.773
